# Polo-like kinase inhibition leads to neuroprotection of neurons bearing alpha-synuclein Lewy body-like inclusions *in vivo*

**DOI:** 10.17912/micropub.biology.001348

**Published:** 2024-10-10

**Authors:** Elizabeth P. Rose, Jovin S. Banga, Vivek K. Unni

**Affiliations:** 1 Jungers Center for Neurosciences Research, Oregon Health & Science University, Portland, Oregon, United States; 2 Department of Neurology, OHSU Parkinson Center, Portland, Oregon, United States

## Abstract

α-synuclein (αSyn) and S129 phosphorylated αSyn (pSyn) define synucleinopathies like Parkinson’s disease (PD). Targeting S129 αSyn kinases, like the Polo-like kinase (PLK) family, could provide a therapeutic strategy to limit degeneration of cells bearing aggregated αSyn inclusions. Using longitudinal *in vivo* multiphoton imaging in mouse cortex after αSyn inclusion induction, we find an increase in cell survival of inclusion-bearing neurons after PLK inhibition. PLK inhibition is associated with increased αSyn levels within inclusions and increased nuclear DNA damage repair markers. Overall, these findings suggest that PLK inhibition may serve as a potential therapeutic strategy for limiting neurodegeneration in PD.

**Figure 1. PLK inhibition protects against neurodegeneration in neurons bearing somatic Lewy inclusions in vivo:**
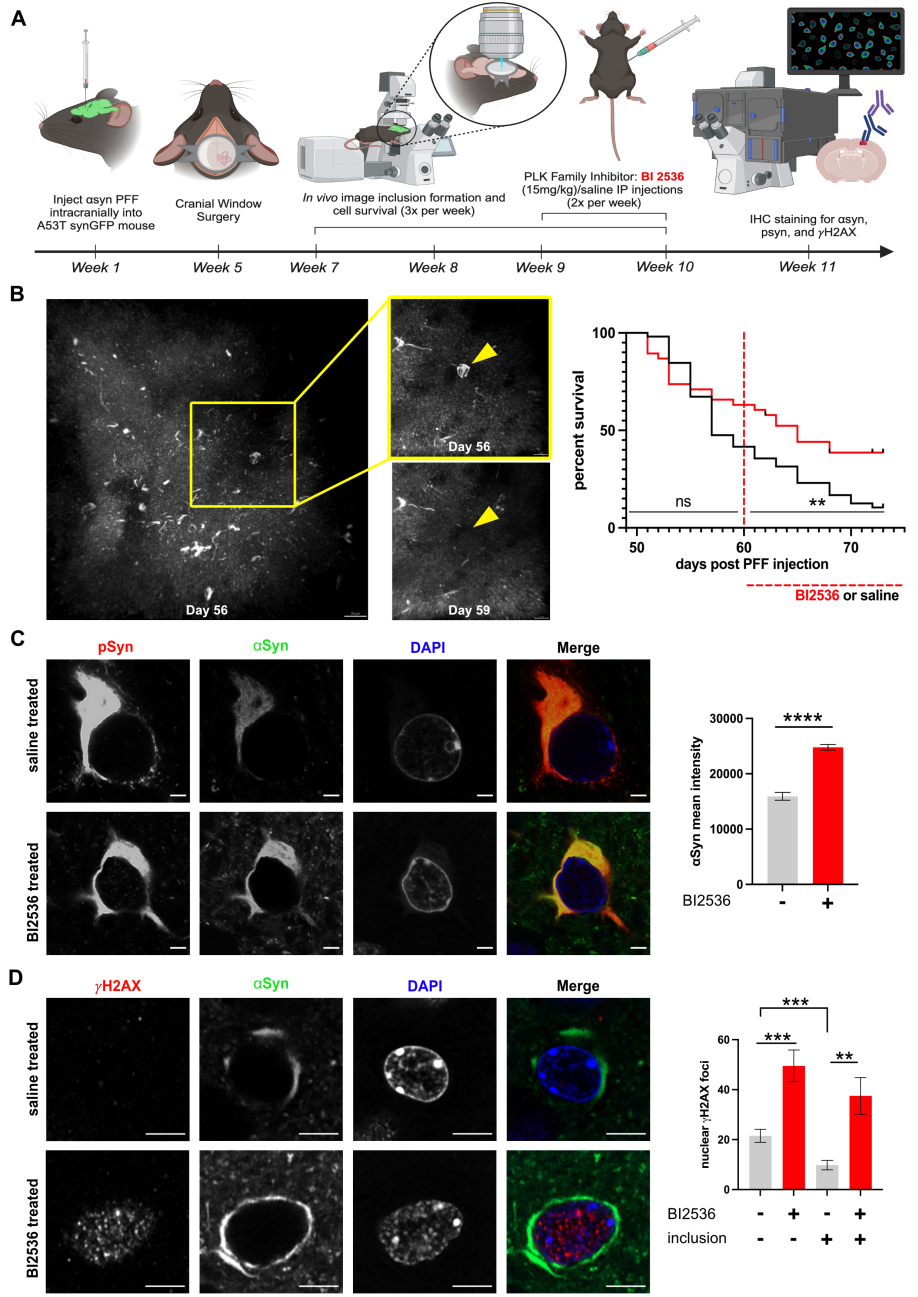
A) Cartoon schematic of experimental protocol to induce Lewy pathology in mouse cortex via intracranial injection of αSyn pre-formed fibrils (PFFs). *In vivo* imaging was performed after cranial window surgery for 4 weeks, imaging each cortical brain region 3x per week. Baseline imaging consisted of 2 initial weeks and then mice were either injected with PLK inhibitor BI2536 or saline 2x per week for 2 weeks. IHC was then performed on dissected brains after drug treatment. Created with BioRender.com. B) Representative images of mouse brain cortex *in vivo* demonstrating loss of cell body bearing αSyn somatic inclusion (yellow arrowhead) from day 55 to day 59 post PFF injection. Scale bar 20mm (LEFT), scale bar 10mm (MIDDLE). Images were taken as separate acquisitions of inclusions (top 21mm z-stack, bottom 36mm z-stack). RIGHT: Survival curve of somatic inclusions across 25 days of longitudinal imaging of cortical regions *in vivo* in mice treated with saline or PLK 1/2/3 Inhibitor BI2536 (15mg/kg) IP injections for 2 weeks starting day 60 post PFF injection. Overall Mantel-cox test p=0.0161. Pre BI2536/saline treatment p=0.1454. Post BI2536/saline treatment p<0.0055. Saline treated group N= 4 animals. BI2536 treated group N=4 animals. 108 inclusions counted. C) BI2536 treatment (15mg/kg IP injections twice per week for two weeks) is associated with no change in pSyn levels, but increased total αSyn levels within PFF-induced aggregated somatic inclusion. Scale bar 2mm. RIGHT: Quantification of synuclein levels within the aggregate. Significant increase of αSyn mean intensity from BI2536 treated mice (24755 ±511.682) compared to saline treated mice (15920 ±707.861)(p<0.0001). No significant difference between pSyn mean intensity from saline treated mice (21766 ±1255.436) and BI2536 treated mice (19698 ±918.248)(p=0.1805), data upon request. No significant difference of volume of the inclusion between saline treated mice (126.6 ±10.803) and BI2536 treated mice (133.6 ±6.606)(p=0.5559), data upon request. N= 5-6 mice in each group, n=143 inclusions. Two-tailed student’s t-test. D) BI2536 treatment is associated with increased DSB levels in PFF-induced cortical Lewy pathology mouse model. Scale bar 5mm. RIGHT: Quantification. Significant increase of nuclear 𝛾H2AX foci in cells without inclusions from BI2536 treated mice (49.55 ±6.334) compared to cells without inclusions from saline treated mice (21.54 ±2.605)(p=0.0007), but no significant difference when compared to cells bearing inclusions from BI2536 treated mice (37.53 ±7.357)(p=0.2178). Significant increase of nuclear 𝛾H2AX foci in BI2536 treated cells bearing inclusions compared to saline treated cells bearing inclusions (9.812 ±1.886)(p=0.0031). Significant decrease of nuclear 𝛾H2AX foci in saline treated cells bearing inclusions compared to cells without inclusions (p=0.0004). N= 5-6 mice in each group, n=349 inclusions. Two-tailed student’s t-test.

## Description

A hallmark of Parkinson’s disease (PD) and other synucleinopathies is α-synuclein (αSyn) aggregation into cytoplasmic inclusions, called Lewy bodies, found within surviving neurons in brain regions susceptible to cell death. It remains unknown, however, how Lewy body pathology, which is enriched in S129 phosphorylated αSyn (pSyn), contributes to cell death. pSyn is the most common post-translational modification αSyn undergoes and is highly enriched in Lewy pathology (Anderson et al., 2006) , but it is unclear whether pSyn promotes or protects against aggregation and/or cell death (Tenreiro et al., 2014) . While S129 phosphorylation has been shown to reduce αSyn fibrillogenesis *in vitro* (Paleologou et al., 2008) , other *in vitro* studies suggest that it can also promote fibrillar aggregation depending on the exact conditions used (Fujiwara et al., 2002; Ma et al., 2016; Samuel et al., 2016) . *In vivo* , studies utilizing a phospho-deficiency approach where S129 is mutated to alanine increased aggregation in drosophila (L. Chen & Feany, 2005), but in rat brain had no effect (McFarland et al., 2009) or reduced (Gorbatyuk et al., 2008) aggregation compared to the phospho-mimic S129D mutation. Another strategy to study the effects of S129 phosphorylation is to modulate the kinases and phosphatases that act at this residue. Several kinase families have been shown to produce pSyn *in vitro* (Ishii et al., 2007; Kawahata et al., 2022; Pronin et al., 2000; Qing et al., 2009; Wu et al., 2020) , but several studies, including our own work, suggest that the Polo-Like Kinase (PLK) family members 1, 2, and 3 (Aubele et al., 2013; Basso et al., 2013; Bergeron et al., 2014; Inglis et al., 2009; Mbefo et al., 2010; Oueslati et al., 2013; Waxman & Giasson, 2011) may be the most important *in vivo* (Weston et al., 2021) . Our previous work demonstrated that genetic knockout of PLK2 led to improved survival of cortical neurons bearing aggregated αSyn Lewy body-like inclusions in mouse cortex *in vivo* (Weston et al., 2021) . Given the attractiveness of kinase inhibitors as potential therapeutic agents, we set out here to test the effect of PLK inhibition on αSyn biology and neurodegeneration.

In order to test how manipulating phosphorylation of αSyn may affect cell death, we measured cell survival of Lewy inclusion-containing neurons longitudinally over 4 weeks, utilizing an *in vivo* multiphoton imaging approach in the A53T Syn-GFP mouse line (Schaser et al., 2020) ( Fig. 1A ). Previous work shows that the GFP-tagging does not detectibly affect synuclein aggregation in this experimental paradigm (Osterberg et al., 2015; Spinelli et al., 2014) . Mouse cortical regions were imaged for a 2-week baseline period, and then for an additional 2 weeks during exposure to the pan-PLK1-3 inhibitor BI2536 or saline control. PLK inhibition started at day 60 after intracortical αSyn preformed fibril (PFF) injection to induce Lewy pathology, a time point we have previously shown leads to robust cortical pathology that can be imaged *in vivo* (Schaser et al., 2020) . No significant differences in the rate of Lewy inclusion-bearing neuron cell death were detected between the two groups of mice during the baseline imaging period before PLK inhibition. However, during the 2-week BI2536 treatment period, we measured an increase in survival rate of Lewy inclusion-bearing cells treated with BI2536 compared to saline control ( Fig. 1B ). These results suggest that acute pharmacologic inhibition of PLK protects against neurodegeneration of neurons bearing Lewy inclusions and extends our previous result in PLK2 KO mice (Weston et al., 2021) , now with a clinically relevant treatment paradigm.

To investigate the effects of PLK inhibition on aggregated αSyn within Lewy pathology, we used fixed tissue immunohistochemstry (IHC) to study neuronal somatic inclusions from mouse cortex after BI2536 or saline treatment after our *in vivo* imaging experiment ( Fig. 1B ) had ended. Interestingly, BI2536 treatment increased the level of total αSyn protein within Lewy inclusions, but had no significant effect on pSyn levels (data reported in figure legend) ( Fig. 1C ). This suggests that PLK1, 2 and 3 are not S129 αSyn kinases for Lewy pathology, but do alter αSyn protein levels within inclusions. Previous work has suggested that PLK inhibition decreases αSyn degradation within aggregates (Oueslati et al., 2013) . Our data are consistent with this result. Because of the growing body of literature linking αSyn to DNA double-strand break (DSB) repair (Arnold et al., 2024; H.-Y. Chen et al., 2024; Rose et al., 2024; Schaser et al., 2019) and previous work showing that PLK2 KO led to increased αSyn in nuclear double-strand break (DSB) repair foci (Weston et al., 2021) , we next tested whether PLK inhibition changes levels of the DSB repair marker, gH2AX. Via IHC, we found that BI2536 treatment caused an increase in gH2AX levels, both in cells with and without somatic Lewy inclusions ( Fig. 1D ). How PLK inhibition leads to an increase in gH2AX is not clear; it could be due to a specific effect on mediators of DSB repair like C-terminal binding protein interacting protein (CtIP), which are known to be phosphorylated by PLK (Barton et al., 2014; Wang et al., 2018) or potentially through its effect to decrease αSyn degradation within cells.

In summary, our *in vivo* multiphoton imaging data show that inhibiting PLK acutely can improve survival of Lewy inclusion-bearing neurons in cortex. This extends our previous work in PLK2 KO mice (Weston et al., 2021) by suggesting that pharmacologic inhibition may have similar effects and be potentially therapeutic. Our previous work in PLK2 KO mice demonstrated that PLK2 was not a Lewy pathology kinase (Weston et al., 2021) and our new data with BI2536, which inhibits PLK1, 2, & 3 further suggests that neither PLK1 or 3 is a Lewy pathology kinase either. We did detect an increase in total aggregated αSyn levels within inclusions after PLK inhibition, however, which is consistent with previous work from Lashuel and colleagues that finds that PLK2 regulates and enhances autophagic clearance of αSyn in a kinase-dependent manner (Oueslati et al., 2013) . More investigation is required to decipher how PLK inhibition leads to increased neuronal survival and whether DSB repair and genomic stability play a role, but it is interesting to speculate that increases in αSyn levels within the inclusion may be mirrored by increases in soluble nuclear αSyn as well that could be promoting more efficient DSB repair.

Together, our data shows that acute PLK inhibition can lead to neuroprotection in a Lewy pathology model and understanding this mechanism better could lead to new treatments for clinically important forms of neurodegenerative disease.

## Methods

**Animals.**

All mice lines were housed in OHSU’s Department of Comparative Medicine (DCM) facilities in a light-dark cycle vivarium. Animals were maintained under *ad libitum* food and water diet. All animal protocols were approved by OHSU IACUC, and all experiments were performed with every effort to reduce the number of animal used and their suffering. The A53T-Syn-GFP mouse line was genetically created (Schaser et al., 2019) and characterized (Schaser et al., 2020) according to our previous research.

**Mouse brain *in vivo* imaging & analysis.**

2 to 3 month-old male and female mice were unilaterally injected with 2.5mL (2mg/mL) of freshly sonicated mouse WT sequence PFFs using the same protocol as we have previously published (Schaser et al., 2019) . Cranial window surgeries were performed 5 weeks post PFF injection according to our previous published protocols. This timeline was chosen based on previous research with this A53T transgenic mouse that has accelerated pathology spread for optimal imaging (Schaser et al., 2020) . Mouse cortex was imaged 3 weeks post cranial window surgery using a Zeiss LSM 7MP multiphoton microscope. Zeiss Zen image acquisition software was used to collect z-stacks from layer 1 to layer 2/3 of the cortex with 3μm intervals at 63x zoom 1. Regions of interest (ROIs) were analyzed in FIJI and inclusions were verified visually for each day of imaging by hand. New inclusions were counted for each day of imaging and scored by hand. No detectible sex differences were observed. Survival curves were created with Prism10 (GraphPad). Cortical regions were imaged for 4 weeks at 3 times per week. After 2 weeks animals were given a 2 week treatment of saline or 15mg/kg BI2536 IP injections twice per week. Animals were sacrificed after 4 weeks of imaging for IHC.

**Immunohistochemistry.**

Mouse Tissue: Brains from 4-5 month old mice were dissected and fixed according to previously published protocols (Schaser et al., 2019) . Brains were sectioned into 50mM coronal slices using a Vibratome LeicaVT1000S. Tissue was fixed, permeabilized, incubated in blocking buffer, stained with primary and secondary antibodies, and mounted as previously published (Schaser et al., 2019) . Imaging was performed on a Zeiss 980 laser-scanning confocal microscope with a 63x oil objective zoom 7.0. Z-stacks of the αSyn inclusion and nucleus with optimal intervals. Analysis was performed in IMARIS using a 3D surface reconstruction of the inclusion and the DAPI channel to create a nuclear mask. We then used IMARIS to quantify the nuclear 𝛾H2AX foci count or αSyn and pSyn levels with an average intensity measurement.

Primary antibodies used were: anti-Syn1, 1:500 dilution, mouse monoclonal, BD Biosciences, cat. 610786; anti-Phospho-Histone H2a.X, 1:500 dilution, rabbit monoclonal, Cell Signaling, cat. 9718; anti-PhosphoS129-Syn EPY1536Y, 1:500 dilution, rabbit monoclonal, Abcam ab51253. Secondary antibodies used were: Alexa Fluor 555 goat anti-mouse, Abcam ab150114; Alexa Fluor 647 donkey anti-rabbit, Jackson ImmunoResearch Laboratories 711605152.

## Reagents

**Table:** 

**Animal**	**Strain Genetic Background**	**Gene with Mutation**	**Genotype**	**Obtained**
A53T-Syn-GFP mouse	A53TGFPXC57/B6	SNCA (αSyn)	A53T ^+^ /A53T ^+^	Created in house

**Table:** 

**Primary Antibody**	**Description**	**Source**
Syn1	Mouse monoclonal, 1:500 dilution, specific to αSyn	BD Biosciences, 610786
𝛾H2AX	Rabbit monoclonal, 1:500 dilution, specific to phosphor-Histone H2a.X	Cell Signaling, 9718
pSyn	Rabbit monoclonal, 1:500 dilution, specific to PhosphoS129-Syn EPY1536Y	Abcam, ab51253

**Table:** 

**Secondary Antibody**	**Description**	**Source**
Mouse IgG	Goat polyclonal, 1:1000 dilution	Abcam ab150114
Rabbit IgG	Donkey polyclonal, 1:1000 dilution	Jackson ImmunoResearch Laboratories 711605152
